# Bi-Allelic TCRα or β Recombination Enhances T Cell Development but Is Dispensable for Antigen Responses and Experimental Autoimmune Encephalomyelitis

**DOI:** 10.1371/journal.pone.0145762

**Published:** 2015-12-22

**Authors:** Nathaniel J. Schuldt, Jennifer L. Auger, Kristin A. Hogquist, Bryce A. Binstadt

**Affiliations:** 1 Departments of Pediatrics, University of Minnesota, Minneapolis, United States of America; 2 Laboratory Medicine and Pathology, University of Minnesota, Minneapolis, United States of America; 3 Center for Immunology, University of Minnesota, Minneapolis, United States of America; Instituto Nacional de Ciencias Medicas y Nutricion Salvador Zubiran, MEXICO

## Abstract

Dual TCRα-expressing T cells outnumber dual TCRβ-expressing cells by ~10:1. As a result, efforts to understand how dual TCR T cells impact immunity have focused on dual TCRα expression; dual TCRβ expression remains understudied. We recently demonstrated, however, that dual TCRβ expression accelerated disease in a TCR transgenic model of autoimmune arthritis through enhanced positive selection efficiency, indicating that dual TCRβ expression, though rare, can impact thymic selection. Here we generated mice hemizygous for TCRα, TCRβ, or both on the C57BL/6 background to investigate the impact bi-allelic TCR chain recombination has on T cell development, repertoire diversity, and autoimmunity. Lack of bi-allelic TCRα or TCRβ recombination reduced αβ thymocyte development efficiency, and the absence of bi-allelic TCRβ recombination promoted γδ T cell development. However, we observed no differences in the numbers of naïve and expanded antigen-specific T cells between TCRα^+/-^β^+/-^ and wildtype mice, and TCR repertoire analysis revealed only subtle differences in Vβ gene usage. Finally, the absence of dual TCR T cells did not impact induced experimental autoimmune encephalomyelitis pathogenesis. Thus, despite more stringent allelic exclusion of TCRβ relative to TCRα, bi-allelic TCRβ expression can measurably impact thymocyte development and is necessary for maintaining normal αβ/γδ T cell proportions.

## Introduction

Clonal selection theory predicted that each lymphocyte should express only one antigen receptor specificity [1]. Indeed, in most lymphocytes, allelic exclusion ensures this singular specificity [2]. Preassembled T cell receptor (TCR) transgenes inhibit V-to-(D)-J rearrangements of the endogenous TCR alleles [2, 3]. It is now known, however, that allelic exclusion is imperfect, resulting in dual TCR-expressing T cells. In mice, an estimated 10% of αβ T cells express dual TCRα chains. TCRβ chain allelic exclusion is more stringent, resulting in only 1–3% of T cells expressing dual β chains [2]. How dual TCR T cells contribute to the function of the immune system remains incompletely understood.

Hypothetically, dual TCR T cells have the potential to permit autoreactive TCRs to escape thymic clonal deletion. Biased competition between the two TCR chains for the pairing chain or for the CD3 complex could result in disproportionate surface expression of the two TCR specificities, potentially masking the presence of a self-reactive TCR if it is the lesser expressed of the two. In fact, dual TCR-expressing T cells possessing an autoreactive TCR have been demonstrated to escape clonal deletion in several TCR transgenic models [4–7]. However, many mouse models with transgene-encoded TCRs harbor an abnormally high number of dual TCR T cells and have an atypical TCR repertoire as a result of unnaturally early TCR expression during thymocyte development. In an effort to study dual TCRα T cells in non-TCR-transgenic animals, TCRα chain hemizygous mice incapable of dual TCRα chain expression were developed and used to study multiple models of immune-mediated disease [8–10]. These studies demonstrated that dual TCRα-expressing T cells were not required for disease development in experimental allergic encephalomyelitis (EAE) or collagen-induced arthritis; TCRα^+/-^ non-obese diabetic (NOD) mice were initially found to be resistant to diabetes, although further investigation found that incomplete backcrossing resulted in the exclusion of essential diabetes genes which may have contributed to the observed resistance to diabetes [8–10]. However, a recent study demonstrated that dual TCRα expression can allow for more efficient positive selection and formation of a broader TCR repertoire [11]. None of these prior studies have investigated the potential contribution of dual TCRβ-expressing T cells in normal or pathogenic immunity.

We recently demonstrated that expression of either dual TCRα or dual TCRβ chains accelerated autoimmunity in the K/BxN TCR transgenic mouse model of arthritis via enhanced positive selection of autoreactive cells [6]. In light of these findings, we sought to investigate whether dual TCRβ expression measurably affects thymocyte development or function in non-TCR-transgenic mice. To that end, we generated mice hemizygous for both TCRα and TCRβ (TCRα^+/-^β^+/-^) on the C57BL/6 background; these mice are unable to generate any dual TCR T cells. We used these “single TCR T cell mice” to investigate the role of dual TCR T cells in thymic selection, T cell homeostasis, TCR repertoire formation, and induced autoimmune disease.

## Materials and Methods

### Mice

Single TCR T cell C57BL/6J mice (TCRα^+/-^TCRβ^+/-^) were generated by breeding C57BL/6 TCRαβ double knockout (DKO) mice (previously generated in our lab through breeding of B6.129S2-*Tcra* ^*tm1Mom*^/J and B6.129P2-*Tcrb* ^*tm1Mom*^/J) with WT C57BL/6 mice [6, 12]. CD45.1 (CD90.2) C57BL/6 were generously provided by Dr. Marc Jenkins. Founder mice for these colonies and CD90.1 C57BL/6J mice were obtained from The Jackson Laboratory. Mice were bred and housed in specific pathogen-free colonies at the University of Minnesota. This study was approved by the University of Minnesota Institutional Animal Care and Use Committee (protocols 1206A15325 and 1503-32409A). The genotypes of mice were confirmed by PCR (primers: B6 TCRα mutant-GATTCGCAGCGCATCGCCTTCTAT, TCRα WT-GATCCTCGGTCTCAGGACAG, and common B6 TCRα-ACAGGAAGGTGAGCCTCAGA; B6 TCRβ mutant forward-CTTGGGTGGAGAGGCRARRC, B6 TCRβ mutant reverse-AGGTGAGATGACAGGAGATC, B6 TCRβ WT forward-TGTCTGAAGGGCAATGACTG, B6 TCRβ WT reverse-GCTGATCCGTGGCATCTATT).

### Bone marrow chimeras

Bone marrow obtained from healthy CD45.1 (CD90.2) C57BL/6 was depleted of T cells and mixed at a 1:1 ratio with healthy T cell-depleted bone marrow collected from either TCRα^+/-^β^+/-^, TCRα^+/-^, TCR β^+/-^, or WT C57BL/6J (all CD45.2, CD90.2) and injected intravenously into lethally irradiated (1000 rad) CD90.1 C57BL/6J mice. Thymus, spleen, and lymph nodes were collected 10 weeks after bone marrow transplantation for further analysis.

### Flow cytometry

The following antibodies were used for flow cytometry: B220 Alexa Flour 700 (RA3-6B2), CD11b Alexa Flour 700 (M1/70), CD11c Alexa Flour 700 (418), CD8 PECy7 (53–6.7), Vα3.2 APC 780 (RR3-16), Vα8.3 APC 780 (B21.14), CD44 PerCPCy5.5 (IM7), NK1.1 PECy7 (PK136), CD 45.1 (104), FoxP3 ef450, and PerCPCy5.5 (FJK-16s) (eBiosciences); CD25 BB515 (PC61.5), B220 V500 (RA3-6B2), CD11b V500 (M1/70), CD3 V500 (500A2), Vβ6 PE (RR4-7), Ly76 V500 (Ter-119), Strepavidin V500, and a mouse Vβ panel FITC (from BD Biosciences); CD3 BV605 (17A2), CD4 PerCP (RM4-5), TCRγδ BV421 (GL3), CD69 BV605 (H1-2F3), Vα2 APC (B20.1), CD4 BV785 (RM4-5), CD8 PE-CF594 (53–6.7), CD117 BV605 (104D2), CD45.1 BV650 (A20), CD90.2 Alexa Flour 700 (30-H12) (Biolegend); Gr-1 V500 (RB6-8C5), CD19 Biotin (1D3), TCRβ APC (H57-597) (Tonbo). CD1d tetramer was kindly provided by Dr. Kris Hogquist. MOG_38-49_:I-A^b^ tetramer was provided by the NIH Tetramer Core Facility. PLP_178-191_:I-A^b^ tetramer was created with the help of Dr. Marc Jenkins and his laboratory at the University of Minnesota as previously described [13]. 2W1S:I-A^b^, B8R:K^b^, and, gp33:D^b^ tetramers were obtained from Drs. Marc Jenkins and Steven Jameson at the University of Minnesota. Specific lymphocyte populations were defined as follows: double negative (DN)1: CD4^-^, CD8^-^, CD44^hi^, CD25^lo^; DN2: CD4^-^, CD8^-^, CD44^hi^, CD25^hi^; DN3: CD4^-^, CD8^-^, CD44^lo^, CD25^hi^, intracellular TCRβ^+^; DN4: CD4^-^, CD8^-^, CD44^lo^, CD25^lo^, surface TCRβ^+^; double positive (DP)_pre_: CD4^+^, CD8^+^, surface TCRβ^+^, CD69^lo^; DP_post_: CD4^+^, CD8^+^, surface TCRβ^+^, CD69^hi^; B cells: B220^+^, CD3^-^; NK cells: CD3^-^, NK1.1^+^; γδ T cells: CD3^+^, γδ TCR^+^, TCRβ^-^; CD4 T cells: CD3^+^, CD4^+^; CD8 T cells: CD3^+^, CD8^+^; NKT cells: CD3^+^, CD1d^+^; Tregs: CD3^+^, CD4^+^, CD25^+^, FoxP3^+^. Tetramer enrichment was performed as previously described [14, 15]. Cells were analyzed using a BD LSR II flow cytometer (BD Biosciences). Data were analyzed with FlowJo Software (Tree Star).

### TCRβ Sequencing

Splenocytes were harvested from 10-week-old WT and single TCR T cell mice. Erythrocytes were lysed using ACK buffer (0.15 M Ammonium chloride, 1 M potassium bicarbonate, 0.1 M EDTA in water) for 5 minutes at room temperature. Splenocytes were then enriched for T cells by using biotin conjugated anti-CD11b (BD Biosciences), anti-CD11c (eBiosciences), anti-B220 (eBiosciences), and anti-biotin magnetic beads (Miltenyi Biotec) before being passed through magnetic columns (Miltenyi Biotec). DNA was then purified from T cell-enriched cells using the DNeasy kit and protocol (Qiagen). Purified DNA was shipped to ImmunoSEQ (Seattle, WA) for TCRβ sequencing. Between 200,000 and 450,000 total sequences were obtained from each sample resulting in between 10,000 and 28,000 unique productively rearranged sequences per mouse. All ImmunoSEQ data are subject to quality control processing in efforts to minimize intrinsic amplification and sequencing errors [16]. The ImmunoSEQ data are contained in S1 Table.

### Immunization

Immunizations consisted of either100 μg B8R_20-27_ (TSYKFESV), 100 μg gp33 (KAVYNFATM) (both provided by S. Jameson), 200 μg MOG_35-55_ (MEVGWYRSPFSRVVHLYRNGK) (Peptides International), or 50 μg PLP_178-191_ (NTWTTCQSIAFPSK) (Peptides International) mixed with 50 μg of poly I:C (Amersham) and 50 μg of α-CD40 (BioXcell), or 100 μg 2W1S (EAWGALANWAVDSA) (GenScript Corp.) mixed with 5 μg LPS (List Biologicals) diluted in 1x PBS to a total volume of 100 μL per injection. Injections were administered retro-orbitally under isoflurane anesthesia.

### EAE Induction

EAE was induced using an emulsion of Freund’s adjuvant plus *Mycobacterium tuberculosis* H37Ra (4 mg/mL) and either 200 μg MOG_35-55_, or 50 μg PLP_179-191_ diluted in phosphate buffered saline (PBS) [17]. Mice were then anesthetized using isoflurane and 200 μL of emulsion was administered subcutaneously dispersed over three locations on the back of the animal. 200 ng of pertussis toxin (List Biological Laboratories) diluted in PBS was administered retro-orbitally immediately following injection of the emulsion and 2 days later. Ketoprofen (5 mg/kg subcutaneously) was administered at the time of immunization and 24 hours later for analgesia. There was no prolonged administration of anti-inflammatory drugs, since they could potentially modify the disease course. Mice were monitored daily for 21 days following immunization. Mice were age- and sex-matched between the experimental groups.

### EAE scoring

EAE scoring was based on a previously published scale ranging from 0–5 [17]. Grade 0, normal mouse good tail tone; grade 1, limp tail; grade 2 limp tail and hind limb weakness (waddling gait); grade 3, partial hind limb paralysis; grade 4, complete hind limb paralysis; grade 5, moribund state. Increments of 0.5 were used for animals falling between grades. Mice were monitored daily. Mice with grades 1–4 were given easier access to food, and grades 3–4 were given moist food as well as subcutaneous fluids (1 mL phosphate buffered saline daily). Grade 4 mice were housed at low density to avoid contact with other mice. Mice were euthanized if they reached grade 5. Euthanasia was achieved by inhalation of carbon dioxide from a compressed gas cylinder followed by cervical dislocation.

### Statistical calculations

Statistical differences between groups were calculated using 2-tailed *t* test or Mann-Whitney nonparametric analysis where indicated (GraphPad). Statistical analysis of EAE over time between groups and TCRβ sequencing data was calculated using two-way ANOVA analysis followed by Bonferroni posttest with a 95% confidence interval calculated using Prism software (GraphPad). The *p* values for Kaplan-Meier survival curves were calculated using log rank test with Prism software (GraphPad). *p* values <0.05 were considered significant.

## Results

**Fig 1:**
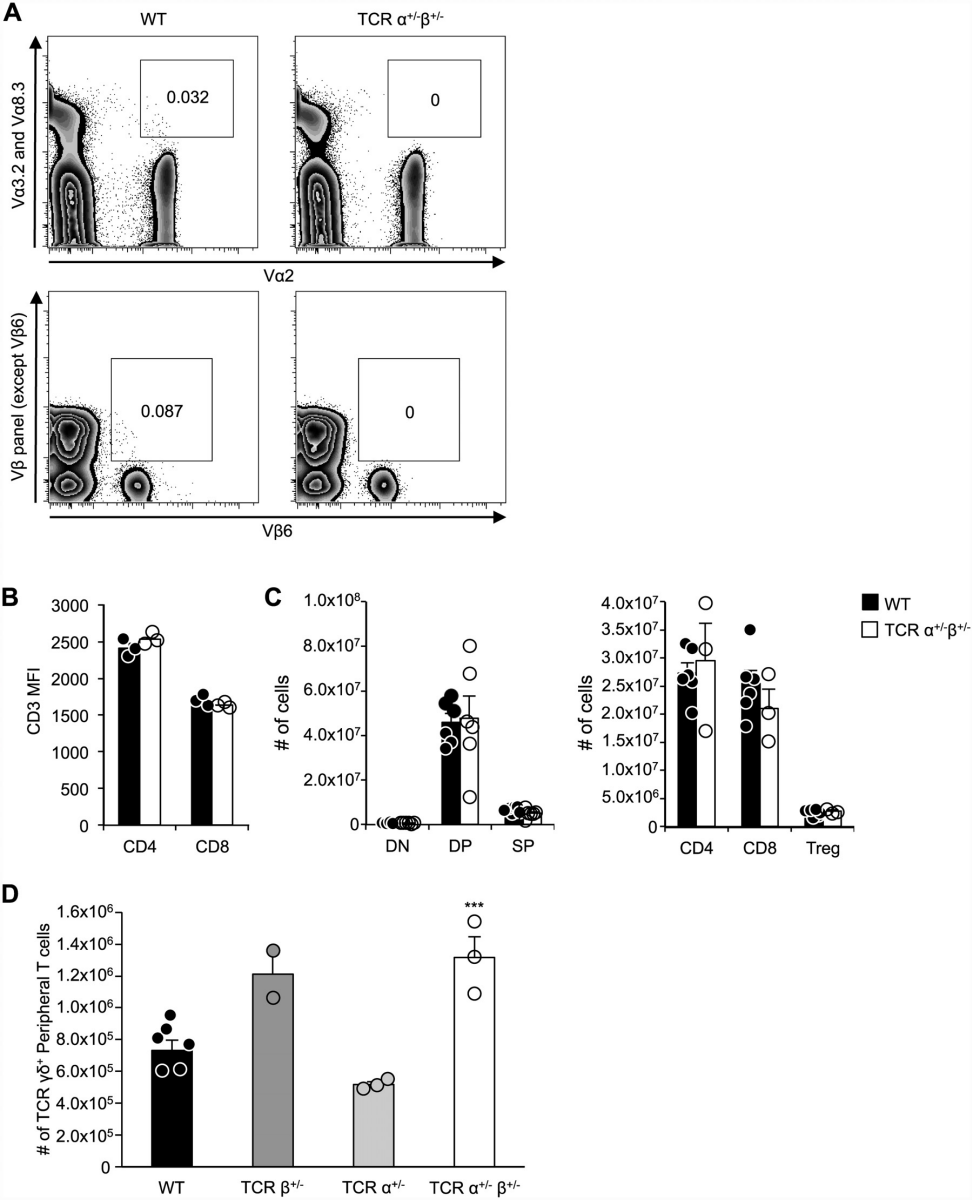
Characterization of single TCR T cell mice. A) Splenocytes were collected from WT and single TCR T cell C57BL/6 mice and analyzed by flow cytometry for co-expression of Vα2 with Vα3.2 or Vα8.3 (top panels) or co-expression of Vβ6 with any of a panel of fourteen other Vβ proteins (bottom panels) in CD3^+^ CD4^+^ T cells. Representative flow plots from three independent experiments show the presence of dual TCRα and β populations in WT (boxed populations in left panels) that are absent in single TCR T cell mice (right panels). B) Flow cytometric analysis of splenocytes from WT and single TCR T cell mice reveals equivalent CD3 expression among CD4^+^ and CD8^+^ T cells. C) Developmental T cell stages (left) and peripheral T cell subsets (right) from WT and single TCR T cell mice were analyzed and enumerated by flow cytometry (n = 6). D) The number of γδ T cells in the lymph nodes and spleen of adult WT or single TCR T cell mice as determined by flow cytometry (B220^-^, CD11b^-^, CD11c^-^, CD3^+^ and TCRγδ^+^). Results shown are mean +SEM (n = 3 for TCRα^+/-^ and TCRα^+/-^ TCRβ^+/-^, n = 2 for TCRβ^+/-^, and n = 6 for WT). Student’s *t*-test was used to determine *p* values; ***p<0.001. Flow cytometry plots are in Log10 fluorescence scale.

Single TCR T cell C57BL/6 mice were generated by breeding TCRα/TCRβ double knockout (DKO) mice to wildtype (WT) mice to obtain mice hemizygous for both alleles (TCRα^+/-^, TCRβ^+/-^) [12, 18]. As predicted, no dual TCRα- or TCRβ-expressing T cells could be identified in these mice (Fig 1A). The level of CD3 cell surface expression on CD4^+^ and CD8^+^ mature T cells was indistinguishable from that in WT mice (Fig 1B). These findings confirmed that single TCR T cell mice lack all dual TCR T cells yet maintain normal cell surface expression of the remaining TCR/CD3 complexes. Furthermore, the absolute numbers of αβ T cells in the double positive (DP), double negative (DN), single positive (SP) thymocyte, and peripheral αβ T cell compartments were comparable in single TCR T cell mice and WT control mice (Fig 1C).

Since recombination of the TCRγ and TCRδ alleles occurs simultaneously and in competition with TCRβ allele rearrangement, we hypothesized that TCRβ^+/-^ thymocytes incapable of bi-allelic TCRβ rearrangement might be more likely to enter the γδ T cell lineage. Indeed, the number of peripheral γδ T cells was increased in TCRα^+/-^ β^+/-^ mice relative to WT mice (Fig 1D), and this trend was recapitulated among the TCRβ^+/-^ mice, but not the TCRα^+/-^ mice.

### TCRβ sequence diversity is broadly similar between WT and single TCR T cell mice

**Fig 2:**
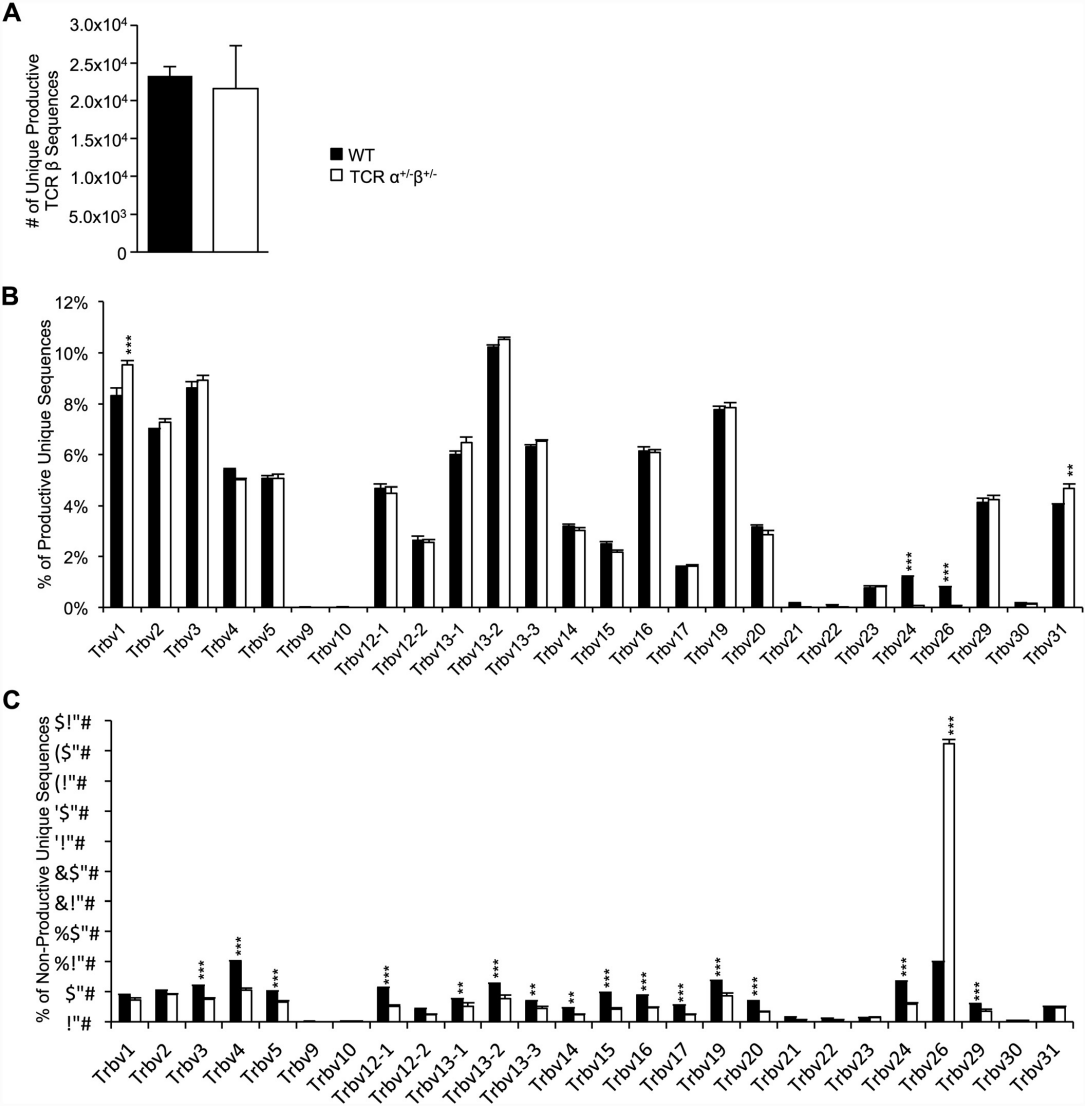
The peripheral TCR repertoire remains diverse despite the absence of bi-allelic TCR rearrangements. TCRβ chain sequencing was performed on DNA purified from T cell-enriched splenocytes from WT and single TCR T cell mice. A) The number of unique productive TCRβ sequences in WT and single TCR T cell mice are similar (n = 3/genotype). Individual *Trbv* usage in B) productively and C) non-productively rearranged WT and single TCR T cell mice is displayed as a percentage of total unique sequences ranging from ~11,000 to ~35,000 sequences per mouse (n = 3/genotype). Results shown are mean +SEM. Two way ANOVA with Bonferroni posttest with a 95% confidence interval was used to determine *p* value. ** and *** indicate *p*<0.01 and *p*<0.001 respectively.

We hypothesized that the lack of bi-allelic TCR gene recombination could result in decreased TCR diversity in single TCR T cell mice. It has recently been demonstrated that some TCRα specificities were observed more commonly among dual TCRα T cells than among single TCRα T cells [11]. To investigate whether a similar phenomenon occurs due to dual TCRβ expression, we collected DNA from T cells harvested from WT and single TCR T cell mice and sequenced the TCRβ chains. We found an equivalent number of unique productively rearranged TCRβ chains in each group indicating TCR diversity remains robust despite the lack of bi-allelic TCR recombination (Fig 2A). There was little overlap between WT and single TCR T cell CDR3α sequences. Altogether, we sequenced 58,931 unique CDR3α sequences obtained from three WT mice and 63,963 unique sequences from three single TCR T cell mice with only 5,772 sequences shared between the two groups. Analysis of the prevalence of individual Vβ genes revealed similar usage between WT and single TCR T cell mice productively rearranged TCRβs with the exception that some of the rare Vβ genes (*Trbv24*, and *Trbv26*) were more prevalent in the WT T cells, and the more common *Trbv1* and *Trbv31* were more often used by T cells from single TCR T cell mice (Fig 2B). Not surprisingly mice lacking a second TCRβ allele had a lower percentage of non-productive sequences than WT mice (6% versus 29% respectively). Non-productive sequences likely mostly existed in the γδ T cell population of single TCR T cell mice. Nonetheless, when we analyzed non-productive TCRβ sequences we observed prevalent usage of *Trbv*26 in sequences obtained from single TCR T cell mice while Vβ usage among the WT mice was more evenly distributed. We similarly analyzed DJβ usage in productive and non-productive sequences. Productive sequences obtained from WT animals trended toward using *Trbj1* genes more often than *Trbj2* while single TCR T cell trended inversely, using *Trbj2* more often than *Trbj1*. The opposite pattern was observed among the non-productive sequences (S1 Fig). Overall, no major shifts in TCRβ chain diversity were observed in the single TCR T cell mice.

### Bi-allelic TCR rearrangement enhances the efficiency of thymocyte development, positive selection

**Fig 3:**
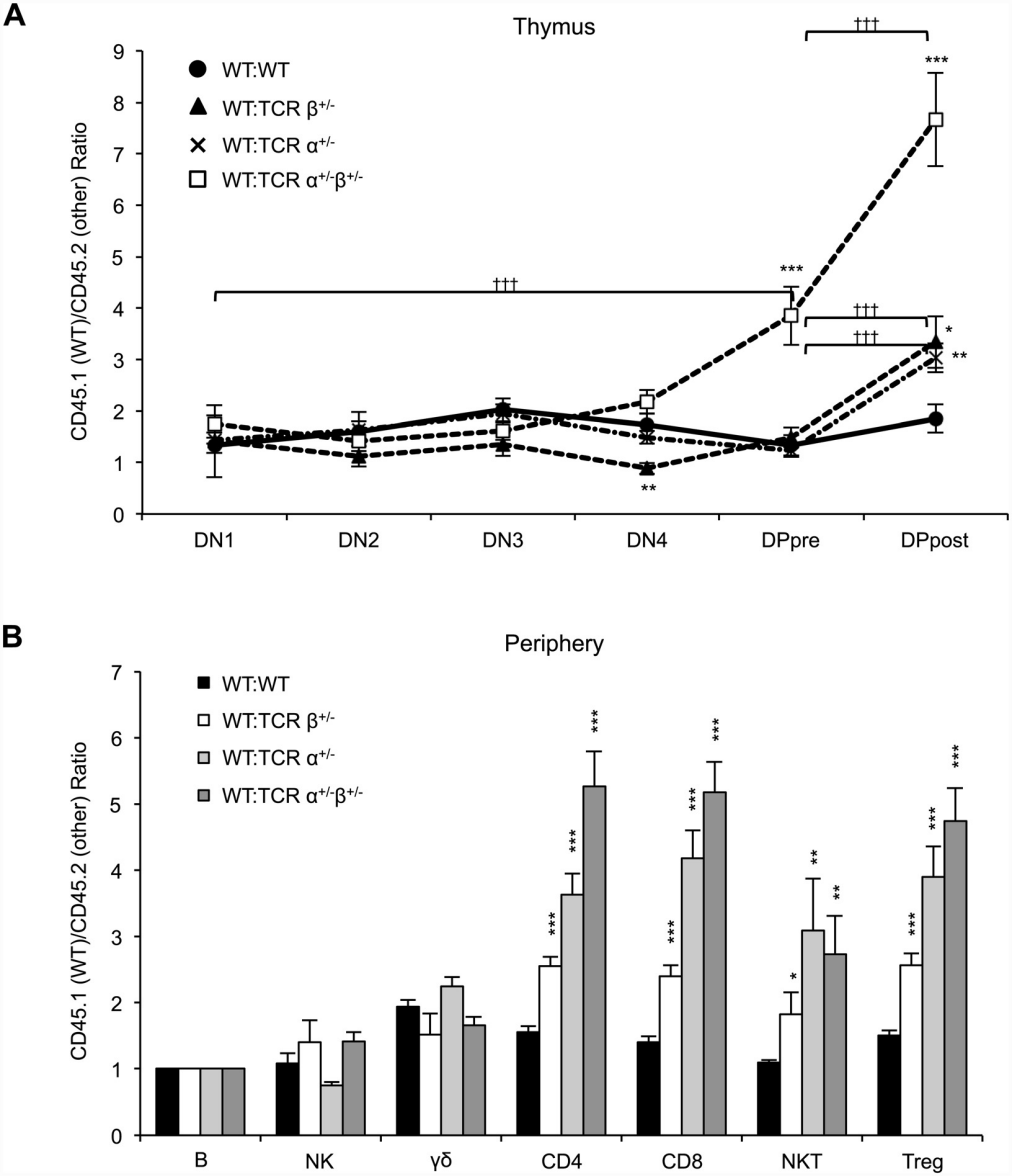
Bi-allelic TCRα and β chain recombination affect thymic selection efficiency. T cell-depleted bone marrow was collected from WT C57BL/6 (CD45.1 and CD90.2) mice and combined with that of either TCRα^+/-^, TCRβ^+/-^, or TCRα^+/-^ TCRβ^+/-^ (all CD45.2 and CD90.2) mice in equal proportions and injected into lethally-irradiated C57BL/6 CD90.1 mice. Thymus, spleen, and lymph nodes were collected 10 weeks following transplantation. The ratios of WT:TCRα^+/-^, WT:TCRβ^+/-^, and WT:TCRα^+/-^ TCRβ^+/-^ (CD45.1:CD45.2) cells were determined by flow cytometric analysis of A) thymocytes at the indicated developmental stages and B) peripheral lymphocyte subsets. All ratios are adjusted to the peripheral B cell CD45.1:CD45.2 ratio in each mouse, as an internal control. The data plotted include the mean and SEM (n = 7–8 mice/group). Student’s *t*-test was used to determine *p* value, relative to the WT:WT control. *, **, and *** indicate *p*<0.05, *p*<0.01, and *p*<0.001 respectively. Student’s *t*-test was used to determine *p* values of CD45.1:CD45.2 ratios pre/post α and β (DP_pre_/DP_post_ and DN1/DP_pre_ respectively). ††† indicates *p*<0.001 for pre/post selection comparisons.

Since bi-allelic rearrangement of TCR loci has been proposed to increase the likelihood of productive gene rearrangement and thereby promote thymocyte survival, we next investigated the effects of bi-allelic TCR gene rearrangement on thymocyte development by pitting WT thymocytes against those from mice hemizygous for the genes encoding TCRα, TCRβ, or both using 50:50 bone marrow chimeras. We found that thymocytes hemizygous for TCRα, TCRβ, or both exhibited an impaired ability to progress beyond the DP pre-selection stage relative to cells of WT origin (Fig 3A). Hemizygosity at both the TCRα and TCRβ loci resulted in more severe impairment than hemizygosity at only one of the loci. We compared the CD45.1:CD45.2 ratios of cells pre- and post-selection to evaluate the efficiency of α and β selection. Cells hemizygous for TCRα, TCRβ, or both were all impeded through α selection, while no differences in β selection efficiency were observed when the DN1 stage was compared to the DN4 stage. However, cells hemizygous for both loci did demonstrate impeded progression when the DN1 stage was compared with the DP_pre_ stage. In the periphery CD4, CD8, NKT, and T_reg_ cell populations all demonstrated skewing toward cells of WT origin over TCRα^+/-^, TCRβ^+/-^, or dual hemizygous cells, although this effect was less pronounced for the TCRβ^+/-^ cells (Fig 3B).

Taken together, our findings indicate that the inability to undergo recombination of the second TCRα locus, TCRβ locus, or both impairs progression through thymocyte development. However, when not in competition with WT cells, the various T cell compartments do eventually equilibrate to the number observed in WT animals, with the exception that γδ T cell numbers are increased in the absence of dual TCRβ expression.

### Mice lacking dual TCR T cells have a normal T cell repertoire and response to foreign antigens

**Fig 4:**
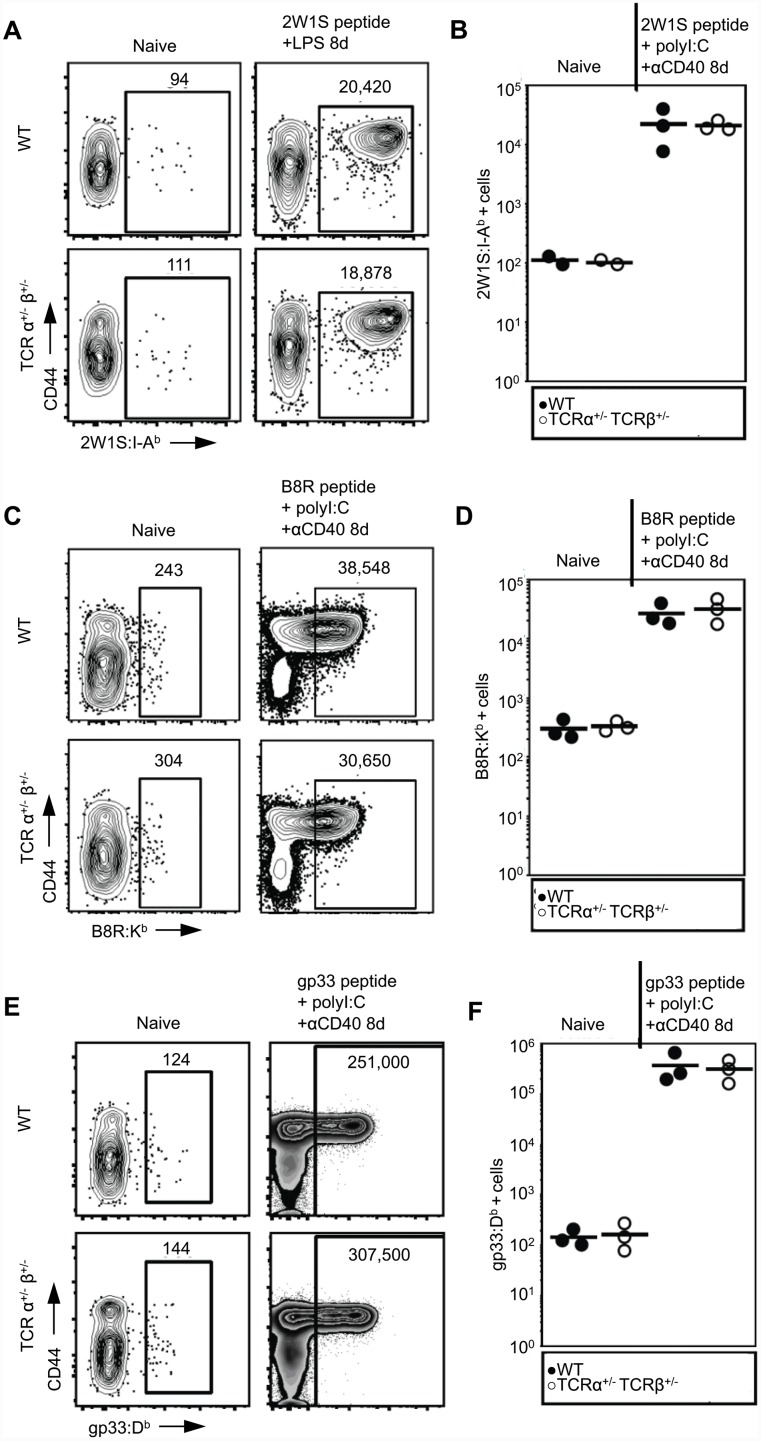
Bi-allelic TCR rearrangements do not impact the foreign antigen TCR repertoire. Splenocytes and lymph node cells were collected from naïve, 2W1S-, B8R-, or gp33-peptide immunized WT and single TCR T cell mice, followed by magnetic tetramer enrichment of antigen-specific populations. Antigen-specific T cells were then stained and analyzed by flow cytometry. A, C, E) Representative plots of naïve (left) and immunized (right) WT and single TCR T cell mice showing 2W1S:A^b^-, B8R:K^b^-, or gp33:D^b^-specific populations respectively. B, D, F) Numbers antigen-specific T cells in naïve and antigen immunized WT and single TCR T cell mice. Each symbol represents one mouse. No statistically significant differences between the two genotypes were present at either of the time points (Student’s t-test). Flow plots are in Log10 fluorescence scale.

We next scrutinized the TCR repertoire of the single TCR T cell mice in response to multiple well-studied foreign antigens, i.e. antigens not under the control of central tolerance [13, 19]. Using a tetramer specific for 2W1S presented by A^b^, we quantified the number of 2W1S-specific CD4^+^ T cells in both naïve single TCR T cell and naïve WT C57BL/6 mice, as well as the expansion of these populations following immunization with the 2W1S antigen. We found no difference in the number of 2W1S-specific CD4^+^ T cells precursors and no defect in the expansion of this population following immunization (Fig 4A and 4B). Likewise, using Class I pMHC tetramers we enumerated CD8^+^ T cells specific for B8R presented by K^b^ and gp33 presented by D^b^ in naïve and immunized single TCR T cell and WT C57BL/6 mice. We observed no difference in the number of CD8^+^ T cells specific for either of these antigens in the two groups. Similarly, no difference in expansion was observed following immunization with either B8R or gp33 (Fig 4C–4F). Together these data demonstrate that for these antigens, the absence of dual TCR T cells does not alter the number of the foreign antigen-specific naïve T cells or their ability to expand following immunization.

### Single TCR T cell mice have normal self-antigen specific T cell precursor numbers and expansion capacity

During negative selection T cells with TCRs that have high affinity for self-antigens are deleted. Dual TCR T cells with a high affinity self-reactive TCR expressed at low levels could theoretically escape negative selection if the other expressed TCR meets the criteria for survival. The lower surface expression of the self-reactive TCR may result in a lower signal during negative selection effectively masking its presence. Once in the periphery this cell could theoretically become activated through the self-reactive TCR, resulting in autoimmunity.

**Fig 5:**
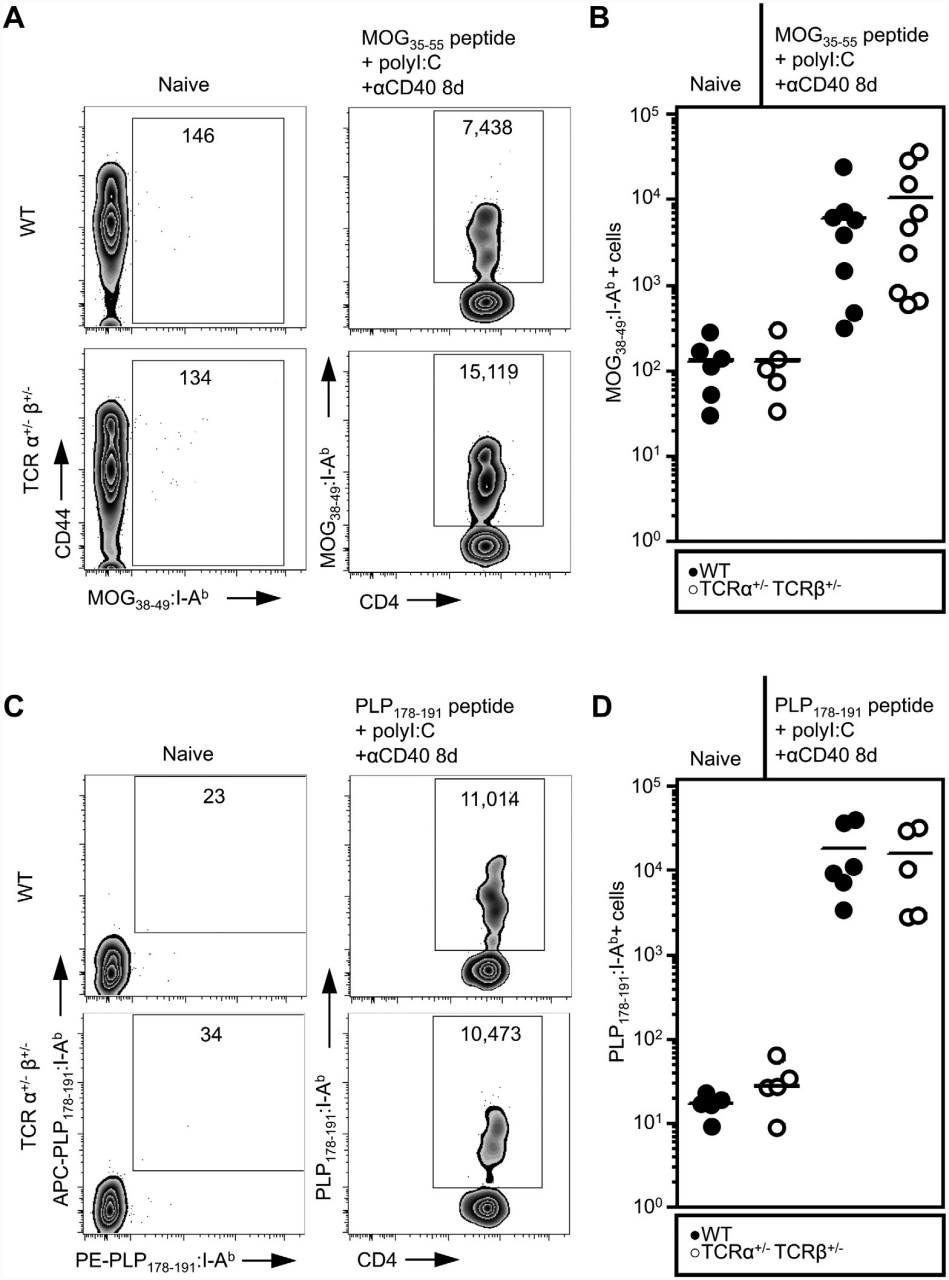
Bi-allelic TCR rearrangements do not alter self antigen-specific T cell repertoire. Splenocytes and lymph nodes were collected from naïve and MOG_35-55_- or PLP_178-191_-immunized WT and single TCR T cell mice. Cells were enriched for antigen-specific populations using tetramers and magnetic cell sorting. Antigen specific T cells were then stained and analyzed by flow cytometry. A) Representative flow cytometry plots and B) numbers of MOG_38-49_:A^b^- specific T cells in naïve (left) and immunized (right) WT and single TCR T cell mice. C) Representative flow cytometry plots and D) number of PLP_178-191_:A^b^-specific populations in naïve (left) and immunized (right) WT and single TCR T cell mice. C) Dual tetramer staining was used to identify the PLP_178-191_:A^b^-specific cells in naïve WT and single TCR T cell mice due to their rarity. In B and D, each symbol represents one mouse. No statistically significant differences between the two genotypes were present at either of the time points (Student’s t-test). Flow plots are in Log10 fluorescence scale.

To begin to test the hypothesis that dual TCR expression impacts autoimmunity we utilized a model of inducible autoimmunity, EAE in C57BL/6 mice. Advantages of the EAE model include the ability to induce disease with antigens that are expressed in the thymus (PLP_178-191_) and therefore impact clonal deletion or with antigens that are less expressed in the thymus (MOG_35-55_) and do not mediate negative selection [20], however, some researchers have more recently reported thymic resident B cell-mediated negative selection of MOG-specific T cells [21]. We first enumerated the PLP- and MOG-specific CD4^+^ T cell precursors in the lymph nodes and spleens of naïve single TCR T cell and WT mice using PLP_178-191_:A^b^ and MOG_38-49_:A^b^ tetramers. Due to the low number of PLP-specific T cells, we utilized a dual tetramer staining technique and counted only cells that stained positive with both PLP_178-191_ tetramers (produced with either streptavidin-APC or -PE). We found no significant difference in the number of MOG- or PLP-specific precursors between WT and single TCR T cell mice (Fig 5A–5D). Additionally, among naïve PLP-specific T cells, we observed no differences in the level of TCRβ, CD3, or CD5 surface expression, nor did we observe a difference in the number of PLP-specific thymocytes or peripheral Tregs (S2A Fig).

We then immunized single TCR T cell mice and WT mice with MOG or PLP in adjuvant. Although the degree of expansion in both the PLP- and MOG-immunized animals varied between animals, presumably due to the low number of precursors [22], we found no significant difference in expansion of the autoreactive T cells between WT and single TCR T cell mice (Fig 5A–5D). In addition, TCRβ, CD3, and CD5 surface expression on PLP-specific T cells and the number of peripheral Tregs were equivalent between PLP-immunized single TCR T cell and WT mice (S2B Fig). Furthermore, immunizing 50:50 bone marrow chimeric mice with MOG_35-55_ resulted in expansion of WT and single TCR MOG-specific T cells in a ratio equivalent to the ratio of the bulk CD4^+^ T cell populations (S3 Fig). Based on these data, we conclude that in C57BL/6 mice with a polyclonal repertoire, dual TCR expression confers no observable benefit to MOG_35-55_ or PLP_178-191_ specific T cells in terms of escaping central tolerance or expanding in response to immunization with the self antigens studied.

### Dual TCR-expressing T cells are not required for initiation of EAE

**Fig 6:**
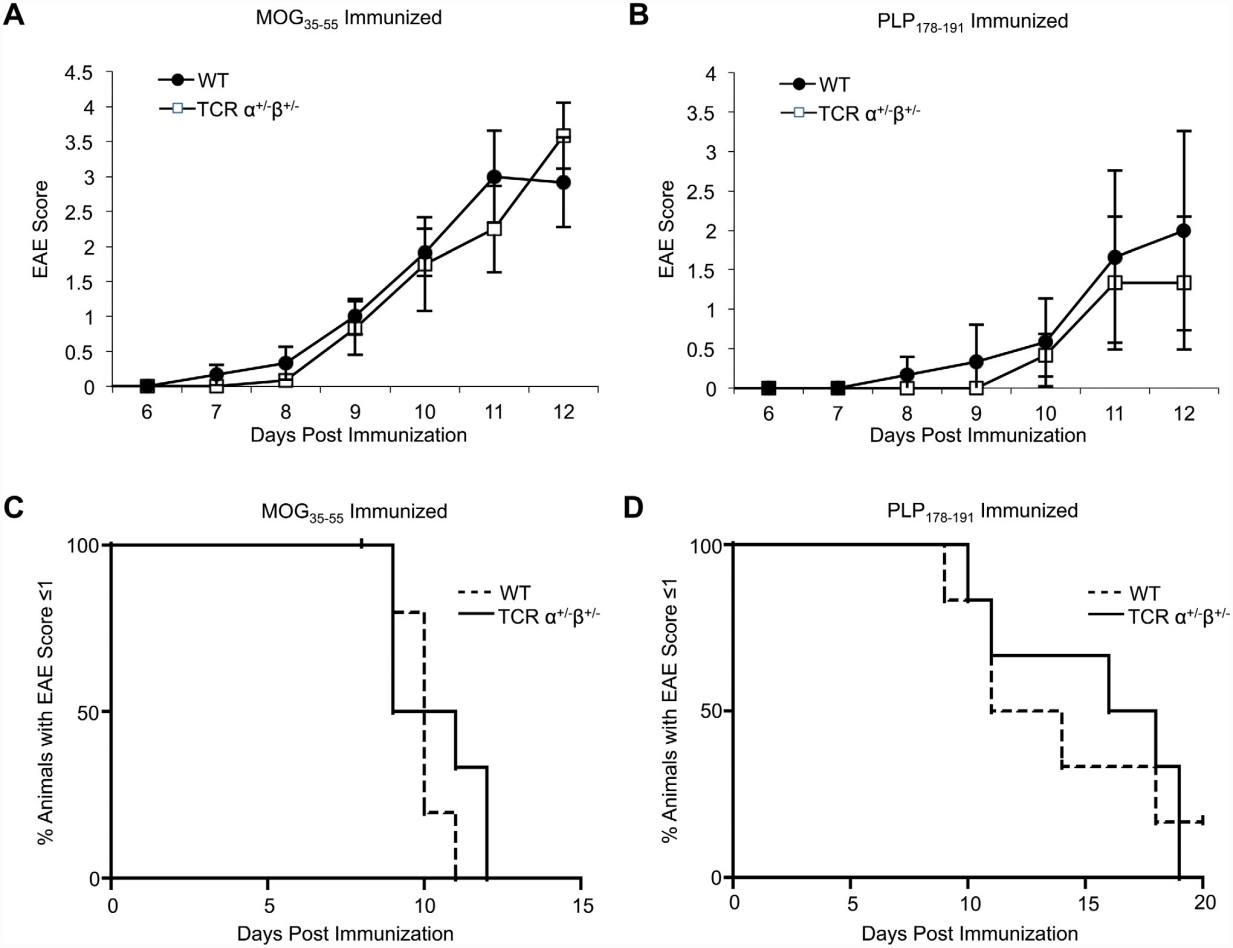
Dual TCR T cells are not required for initiation of EAE. EAE was induced in WT and single TCR T cell mice by immunization with 200 μg MOG_35-55_ or 50 μg PLP_178-191_ plus adjuvant. EAE severity scores were determined daily for up to 21 days post injection. A and B) Average EAE scores of MOG_35-55_- and PLP_178-191_- induced EAE in WT and single TCR T cell mice over time. Data points are depicted as the mean +/- SEM. One-way ANOVA was used to determine *p* values (n = 12-15/group). C and D) Percentage of WT or single TCR T cell mice with an EAE score equal to or greater than 1 in MOG_35-55_- and PLP_178-191_- induced EAE. The *p* values for Kaplan-Meier curves were calculated using log rank test with Prism software (GraphPad); no significant statistical difference was observed.

It remained possible that the pathogenic potential of the expanded autoreactive T cells could differ between WT and single TCR T cell mice. To address this possibility, we induced EAE in single TCR T cell and WT mice using MOG_35-55_ or PLP_178-191_. For both antigens, WT and single TCR T cell mice exhibited similar disease onset, severity, and incidence (Fig 6A–6D). These findings agree with a previous report demonstrating that dual TCRα T cells are not required for EAE [8], and extend them to exclude a requisite role for dual TCRβ expression in this disease model.

## Discussion

Since the discovery that TCR allelic exclusion is imperfect [23], the potential role that dual TCR T cells play in immunity has been of interest, particularly with respect to their potential role in autoimmune disease pathogenesis. TCR transgenic mouse models have demonstrated that dual TCR expression can permit otherwise excluded/non-selected TCRs to enter the peripheral T cell pool, essentially as stowaways. This effect of dual TCR expression has been hypothesized to expand the peripheral TCR repertoire by including both foreign- and self-recognizing TCRs that otherwise would not survive thymic selection [6, 11, 24, 25]. We and others [11] have now demonstrated that either dual TCRα or dual TCRβ expression can allow thymocytes to progress through thymic development more efficiently. Thus, it has been hypothesized that the potential detrimental effect of allowing some self-reactive T cells into the periphery may be outweighed by the increased efficiency of thymocyte development that dual TCR expression confers [11].

Bi-allelic TCRα chain rearrangement has recently been demonstrated to enhance positive selection resulting in increased allo- and autoreactive TCR specificities [11]. Our data supports this finding by demonstrating that thymocytes capable of bi-allelic TCRα chains rearrangement out-compete thymocytes with only one functional TCRα allele. We expand on this finding by demonstrating that bi-allelic TCRβ chain rearrangement also plays a significant role in thymocyte development. We found that, when placed in competition, WT cells outnumbered single TCRβ cells in the post-selection compartment. We suspect that the lack of bi-allelic TCRβ rearrangements likely delayed progression of single TCRβ cells through the DN2 and DN3 stages, but this effect did not become measurably evident until several rounds of expansion had occurred. Sensitivity limitations of the assay and difficulty isolating the DN4 population with high fidelity may have contributed to our inability to determine impacts of hemizygosity during early stages of thymocyte development. Our analysis of the peripheral T cell populations indicated that bi-allelic rearrangement of the TCRβ locus indeed impacts the mature T cell pool, however, to a lesser extent than does bi-allelic TCRα rearrangement. The reason that dual TCRα rearrangement has a greater impact on T cell maturation than dual TCRβ rearrangement might relate to the timing and sequence of recombination. During TCRβ recombination, only one allele recombines at a time. Only after failure of the first locus to rearrange productively does the second locus then begin to rearrange. In a TCRβ^+/-^ T cell, there is no opportunity for a second TCRβ recombination event; thus, failure of rearrangement is expected to lead to death of that cell or commitment to the γδ lineage. Therefore, when WT and single TCRβ cells are in competition, the WT cells only gain an advantage when the first recombination event is unsuccessful. In contrast, during TCRα recombination, both alleles recombine simultaneously. At this stage WT cells may have an even greater advantage over TCRα^+/-^ cells since the WT cells have the opportunity to produce a functional TCRα chain twice as often. These considerations might explain why, when placed in competition, single TCRα expression resulted in more skewing toward cells of WT origin than did single TCRβ expression. Notably, however, a measurable additive effect occurred when TCRα^+/-^TCRβ^+/-^ cells were in competition with WT cells resulting from the combination of delays in development at two separate stages.

Although the lack of bi-allelic TCR allele rearrangement clearly resulted in less efficient T cell progression through thymic development, it did not impact the final overall size of the mature αβ T cell compartments. When not in competition with cells capable of bi-allelic TCR allele rearrangements single TCR T cells eventually produce equivalent numbers of T cells at every stage of development with no obvious detriment to the function of the immune system or health of the animal. It therefore seems unlikely that increased efficiency of thymic selection is the critical evolutionary advantage conferred by incomplete allelic exclusion. Rather, it seems more likely that the persistence of dual TCR expression over evolutionary time may reflect an evolutionary advantage resulting from the broadening of the immune repertoire by dual TCR T cells, an effect that may outweigh the theoretical disadvantage of selecting more autoreactive specificities, especially when one considers the existence of peripheral tolerance mechanisms [11, 24].

The increased frequency of γδ T cells we observed in single TCR T cell mice may deserve additional consideration. The TCRγ and δ loci are in competition with the TCRβ loci to be the first to recombine a functional receptor (γδ TCR or pre-TCR, respectively) and to establish the cell’s lineage. It was therefore not surprising to us that the inability to undergo bi-allelic TCRβ rearrangement influenced αβ/γδ T cell balance in single TCR T cell mice, favoring γδ loci rearrangement and resulting in an increased number of γδ T cells in the periphery. How this increased number of γδ T cells might impact immune function remains unknown. γδ T cells have been shown to be capable antigen presenting cells, potent pro-inflammatory cytokine producers, and even to possess immunoregulatory effects [26]. Their role in EAE is controversial with some models indicating a pathogenic role, while others demonstrate a protective role [27]. C57BL/6 mice lacking γδ T cells exhibited reduced incidence and severity of EAE [27, 28]. Therefore, it is possible that in our single TCR T cell mice, the increased size of the γδ T cell population could result in more severe EAE potentially counterbalancing a reduction in disease severity resulting from the lack of dual TCR (α/β) T cells. Furthermore, γδ T cells respond to *M*. *tuberculosis*, a key component of the complete Freund’s adjuvant used to induce EAE [29]. Further investigation is required to determine whether the expanded γδ T cell population in the single TCR T cell mice measurably affects EAE progression.

We sequenced the TCRβ repertoire of WT and single TCR T cell mice to determine if there was an impact on TCR diversity. We observed very little overlap in the CDR3α sequences between WT and TCRα^+/-^β^+/-^ T cells, deep sequencing would need to be undertaken to determine if there are sequences that are specifically excluded in the setting of TCRα and β hemizygosity. However, when we analyzed Vβ usage in productively rearranged sequences we found that the two groups differed in their usage of a few Vβs; most notably the rare Vβ genes, *Trbv24*, and *Trbv26*, were more common in WT T cells. Other rare Vβ genes also appeared to similarly trend, though they did not achieve significant differences. Why these gene segments are rarely used is not clear. It is possible that they are predisposed to create weakly- and/or self-reactive TCRs resulting in poorer positive selection. If true, this could indicate that the capacity to express dual TCRs indeed expands the peripheral TCR repertoire. Interestingly, *Trbv26* was the most commonly observed Vβ in non-productive sequences obtained from single TCR T cell mice. The reasons for this finding are unknown but could relate to several variables including but not limited to: an increased likelihood of generating stop codons relative to other Vβs, order of Vβ gene usage during recombination, or γδ TCR recombination rate and timing relative to β recombination. Analysis of DJβ usage showed a trend toward DJβ1 gene usage over DJβ2 genes in WT mice, while the inverse trend was observed in single TCR T cell mice. These finding can be predicted on first principles. DJβ genes are used sequentially during recombination (first DJβ1 genes then DJβ2 genes) therefore; cells that possess only one TCRβ allele (single TCR T cell mice) have no option but to use the DJβ2 genes when attempts at DJβ1 recombination fail, while WT cells capable of bi-allelic recombination can either undergo recombination of the other allele’s DJβ1 segment or use the DJβ2 segment on the first allele. Importantly, the decreased usage of these rare Vβ regions in the single TCR T cell mice did not measurably reduce the number of total T cells or the number of naïve antigen-specific T cells for the antigens we tested. It is worth noting that CD4^+^ T cells responding to MOG_35-55_ predominately use Vβ8.2 (*Trbv13*.*2*), with some usage of Vβ1 (*Trbv5*), Vβ14 (*Trbv31*), and Vβ15 (*Trbv20*) [30, 31]. We found all of these to be either equally common among single TCR T cell and WT mice ((Vβ8.2 (*Trbv13*.*2*), Vβ1 (*Trbv5*), and Vβ15 (*Trbv20*)) or slightly more common in single TCR T cell mice (Vβ14 (*Trbv31*)). It is unknown which Vβ gene is most prominent in C57BL/6 animals immunized with PLP_178-191_. It remains possible that other models of autoimmunity or other strains of mice may rely more heavily on rarely-used Vβ sequences and that a role for dual TCR expression in such models could be demonstrated.

While we and others have described subtle differences in the TCR repertoire of TCRα^+/-^ [11, 25] or TCRα^+/-^β^+/-^ mice, we observed no demonstrable effect on the number of naïve or expanded T cells specific for the foreign and self antigens we tested. In contrast, other investigators have demonstrated a decreased frequency of naïve alloantigen-specific CD4^+^ T cells in TCRα^+/-^ mice [11]. This prior report also found that the frequency of naïve CD4^+^ T cells specific for MOG_38-49_:A^b^ was subtly reduced in TCRα^+/-^ mice [11], whereas we observed that genetic exclusion of all dual TCR T cells had no impact on the number of naïve or expanded MOG_38-49_:A^b^-reactive T cells. The explanation for this minor discrepancy with respect to naïve MOG-specific cells is unclear, but may be due to the prior study reporting frequency of cells while we determined the absolute numbers of cells. Considering our data and those prior data together, it is reasonable to conclude that dual TCR expression may impact the number of naïve T cells specific for certain antigens, particularly alloantigens, but not for most foreign or self antigens, and that the capacity for these latter populations to expand following stimulation is unimpaired in mice lacking dual TCR T cells [11, 25].

Multiple TCR transgenic mouse models of autoimmune disease have implicated dual TCR T cells as key initiators of autoimmunity. However, results from TCR transgenic models must be carefully interpreted as their TCR repertoires are not representative of a natural polyclonal TCR repertoire. Here we have demonstrated that mice lacking any dual TCR T cells, but otherwise possessing a polyclonal T cell repertoire, retain a normal number of autoreactive T cells and remain capable of developing induced autoimmunity. This finding agrees with previously published studies indicating no requirement for dual TCRα T cells in initiation of EAE and expands on those findings to exclude a role for dual TCRβ T cells in EAE as well [8]. It is important to note that immunization with self-peptide plus potent adjuvant creates a highly inflammatory environment capable of activating a small number of autoreactive effector T cells and bypassing normal peripheral tolerance mechanisms [32, 33]. Therefore, induced autoimmunity models do not necessarily recapitulate the immune environment of spontaneous autoimmune diseases. It therefore remains possible that dual TCR T cells could play a role in autoimmune diseases dependent upon rarer TCR variable regions or in the context of impaired tolerance mechanisms, such as in models of spontaneous autoimmune disease like the non-obese diabetic (NOD) mouse [34]. Indeed, lack of dual TCRα T cell has previously been demonstrated to impact both insulitis and incidence of diabetes in the NOD model of type 1 diabetes [8]. However, a follow up study by the same authors indicated that these effects could have been due to incomplete backcrossing resulting in the exclusion of some genes that are essential for induction of diabetes [10]. Our future experiments aim to eliminate dual TCRα and β T cells in the NOD model with complete backcrossing to explore how dual TCR T cells impact spontaneous autoimmune disease models.

## Supporting Information

S1 Fig DJβ2 gene usage is more common in cells from single TCR T cell mice relative to WT. DJβ gene usage was analyzed from TCRβ sequences obtained from T cell enriched cells of WT or single TCR T cell origin. A comparison of Dβ A) and individual Jβ B) gene usage in unique productive TCRβ sequences from WT and single TCR T cell mice. A comparison of Dβ C) and Jβ D) gene usage in unique non-productive TCRβ sequences obtained from WT and single TCR T cell mice. Results shown are mean +SEM. Two-way ANOVA with Bonferroni posttest with a 95% confidence interval was used to determine *p* value. *, ** and *** indicate *p*<0.05, *p*<0.01 and *p*<0.001 respectively.
(PDF)

S2 Fig PLP178-191 specific T cell responses are similar in WT and single TCR T cell mice. Thymocytes and peripheral lymphocytes were collected from A) naïve and B) PLP178-191-immunized WT and single TCR T cell mice. Cells were enriched for PLP178-191- specific T cells using tetramers and magnetic sorting technology. A) Comparison of the number of PLP tetramer-specific CD4+ T cells in the thymus and Tregs. In the periphery were not significantly different between naive WT and single TCR T cell mice. The levels of TCR, CD3, and CD5 expression were also equivalent between naive WT and single TCR T cell mice as measured by mean fluorescent intensity (MFI) by flow cytometry. B) Comparison of PLP178-191-immunized WT and single TCR T cell mice also showed no difference in the number of peripheral PLP-specific Tregs or surface expression of TCR, CD3, and CD5 on PLP specific cells as determined by MFI and flow cytometry. Data are depicted as mean +SEM (n = 3/group). Mann-Whitney nonparametric anaylsis was used to determine *p* values.
(PDF)

S3 Fig The MOG-specific T cell populations of WT:single TCR T cell bone marrow chimeras are not enriched for WT cells relative to the rest of the CD4^+^ T cell population. WT:TCRα^+/-^ β^+/-^ bone marrow chimeras were generated as in Fig 3 and immunized after reconstitution with MOG_35-55_ peptide and adjuvant. Spleen and lymph nodes were harvested 7 days post immunization and the MOG-specific CD4^+^ T cell population was analyzed to determine the CD45.1(WT):CD45.2(TCRα^+/-^ β^+/-^) ratio and compared to the non-MOG-specific CD4^+^ t cell population. Student’s *t*-test was used to determine statistical significance.
(PDF)

S1 Table ImmunoSeq Data. The table contains two sheets, one showing productive rearrangements and the other non-productive TCRβ rearrangements. The number of times each sequence appears is indicated in the table. Data are shown for three WT and three single TCR T cell C57BL/6 mice.
(XLSX)
