# Supplementary figures and images for: Bi-Allelic TCRα or β Recombination Enhances T Cell Development but Is Dispensable for Antigen Responses and Experimental Autoimmune Encephalomyelitis
Source: PLoS One. 2015 Dec 22;10(12):e0145762. doi: 10.1371/journal.pone.0145762 (PMC4687847; doi:10.1371/journal.pone.0145762)

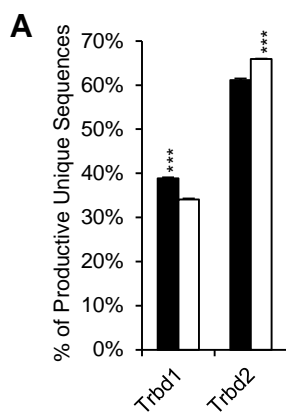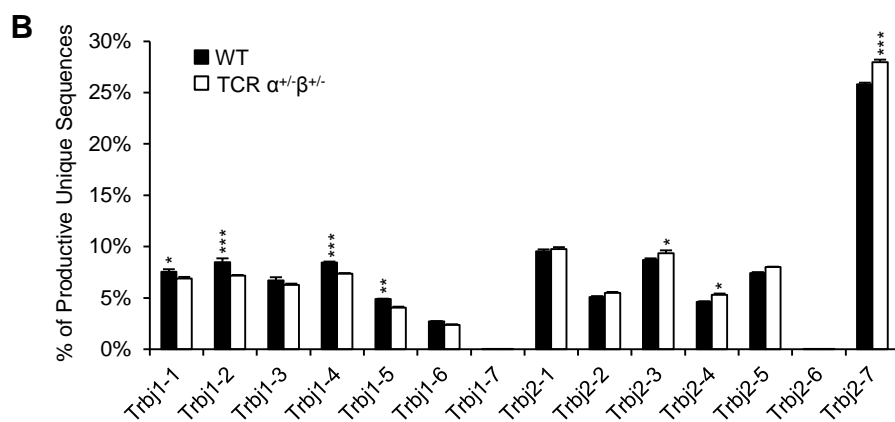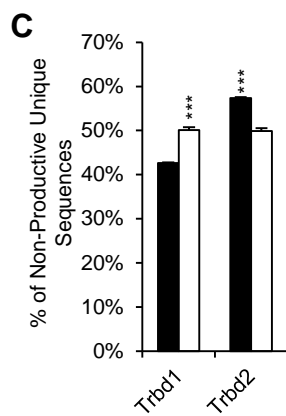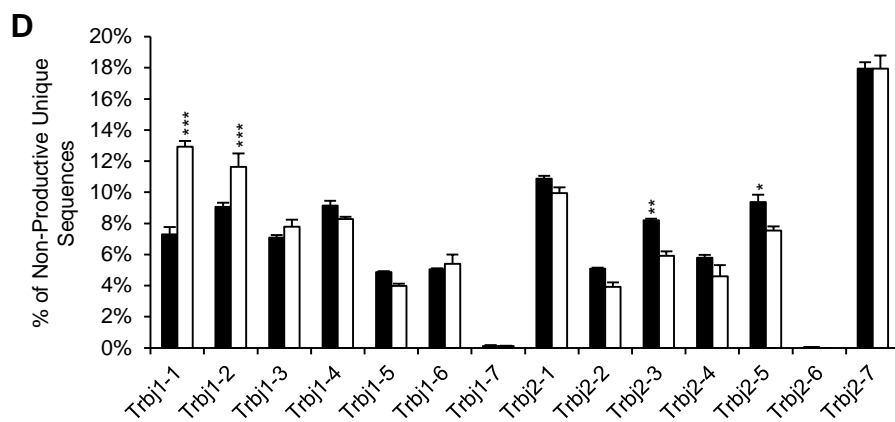

Supplement: S1 Fig — DJβ gene usage was analyzed from TCRβ sequences obtained from T cell enriched cells of WT or single TCR T cell origin. A comparison of Dβ A) and individual Jβ B) gene usage in unique productive TCRβ sequences from WT and single TCR T cell mice. A comparison of Dβ C) and Jβ D) gene usage in unique non-productive TCRβ sequences obtained from WT and single TCR T cell mice. Results shown are mean +SEM. Two-way ANOVA with Bonferroni posttest with a 95% confidence interval was used to determine p value. *, ** and *** indicate p<0.05, p<0.01 and p<0.001 respectively. (PDF) [file pone.0145762.s001.pdf]

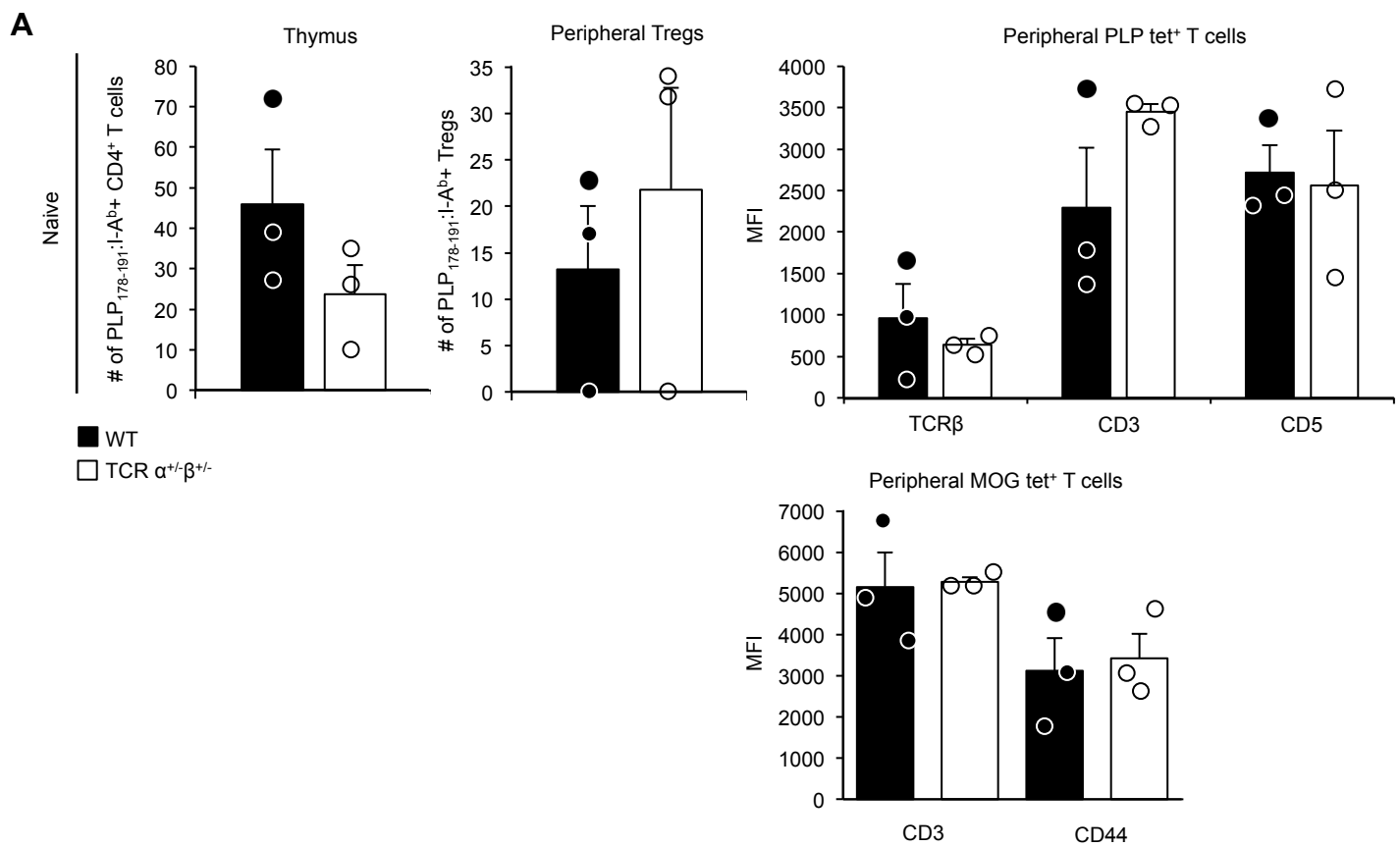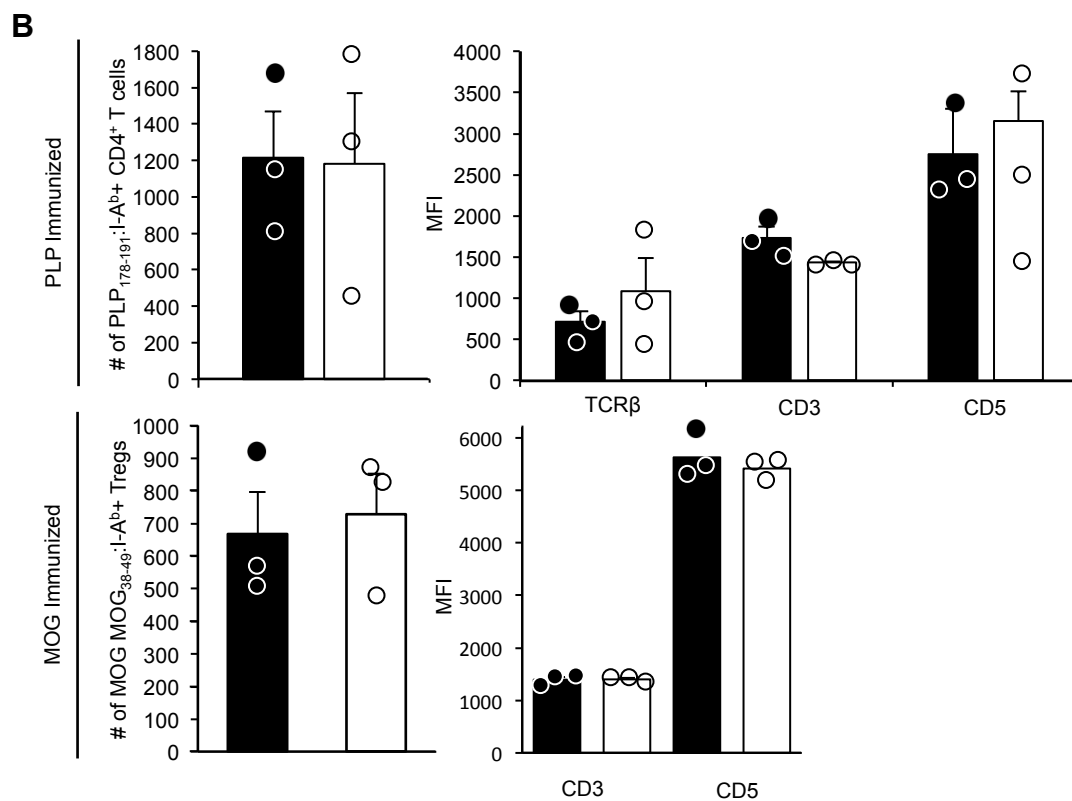

Supplement: S2 Fig — Thymocytes and peripheral lymphocytes were collected from A) naïve and B) PLP178-191-immunized WT and single TCR T cell mice. Cells were enriched for PLP178-191- specific T cells using tetramers and magnetic sorting technology. A) Comparison of the number of PLP tetramer-specific CD4+ T cells in the thymus and Tregs. In the periphery were not significantly different between naive WT and single TCR T cell mice. The levels of TCR, CD3, and CD5 expression were also equivalent between naive WT and single TCR T cell mice as measured by mean fluorescent intensity (MFI) by flow cytometry. B) Comparison of PLP178-191-immunized WT and single TCR T cell mice also showed no difference in the number of peripheral PLP-specific Tregs or surface expression of TCR, CD3, and CD5 on PLP specific cells as determined by MFI and flow cytometry. Data are depicted as mean +SEM (n = 3/group). Mann-Whitney nonparametric anaylsis was used to determine p values. (PDF) [file pone.0145762.s002.pdf]

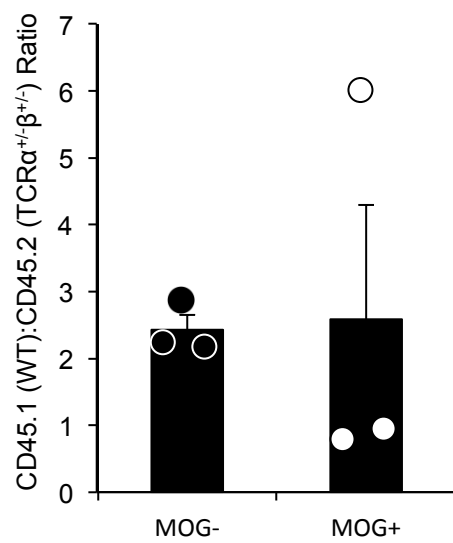

Supplement: S3 Fig — WT:TCRα+/- β+/- bone marrow chimeras were generated as in Fig 3 and immunized after reconstitution with MOG35-55 peptide and adjuvant. Spleen and lymph nodes were harvested 7 days post immunization and the MOG-specific CD4+ T cell population was analyzed to determine the CD45.1(WT):CD45.2(TCRα+/- β+/-) ratio and compared to the non-MOG-specific CD4+ t cell population. Student’s t-test was used to determine statistical significance. (PDF) [file pone.0145762.s003.pdf]
